# Ultraviolet components offer minimal contrast enhancement to an aposematic signal

**DOI:** 10.1002/ece3.6969

**Published:** 2020-11-05

**Authors:** Justin Yeager, James B. Barnett

**Affiliations:** ^1^ Biodiversidad Medio Ambiente y Salud Universidad de Las Américas Quito Ecuador; ^2^ Department of Psychology, Neuroscience & Behaviour McMaster University Hamilton ON Canada

**Keywords:** aposematism, coloration, Dendrobatidae, sexual signals, UV reflection, visual modeling

## Abstract

Aposematic and sexual signals are often characterized by bright, highly contrasting colors. Many species can see colors beyond the human visible spectrum, and ultraviolet (UV) reflection has been found to play an important role in communication and sexual selection. However, the role of UV in aposematic signals is poorly explored. Poison frogs frequently produce high‐contrast signals that have been linked to both aposematism and intraspecific communication. Yet despite considerable efforts studying interspecific and intraspecific diversity in color, poison frogs are not known to perceive UV, and UV reflection of the integument has not been described. We report UV‐reflective spots in a population of *Oophaga sylvatica* and quantify the effect of UV on visual contrast with models of avian vision. We found that the frogs are highly contrasting, but UV had a minimal effect on signal saliency. These data highlight the importance of considering UV reflectance within aposematic signals, but that UV should not necessarily be regarded as an independent signal.

## Graphical abstract

Many species can see colors beyond the human visible spectrum, and UV reflection has been found to play an important role in communication and sexual selection. However, the role of UV in aposematic signals is poorly explored. We found that the frogs are highly contrasting, but UV had a minimal effect on signal saliency.

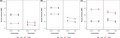

## 1 INTRODUCTION

Color patterns can serve a variety of antipredator functions (Cuthill et al., 2017). Cryptic coloration can reduce the likelihood of detection and subsequently lower the risk of predation (Cuthill, 2019; Merilaita et al., 2017). Meanwhile, aposematic coloration reduces predation risk by utilizing memorable phenotypic traits in conjunction with a form of defense which renders prey unpalatable (Caro & Ruxton, 2019; Stevens & Ruxton, 2012).

Brighter and more strongly contrasting colors are thought to be more effective and memorable aposematic signals (Aronsson & Gamberale‐Stille, 2009; Gamberale‐Stille, 2001; Halpin et al., 2020; Prudic et al., 2006; Stevens et al., 2010). As such, certain pattern components frequently reoccur in unrelated taxa, notably longwave colors such as red and yellow, which are often combined with black. Such combinations create high achromatic (luminance) and chromatic (hue) contrast both between pattern components and against the background (Aronsson & Gamberale‐Stille, 2009, 2012).

High visual contrast can also be created with shortwave colors such as blue and ultraviolet (UV). UV‐sensitive vision is present among taxonomically diverse groups, including birds, lizards, fish, butterflies, and jumping spiders (Cronin & Bok, 2016), and as such may be incorporated into salient signals including sexual communication and aposematism (Andersson et al., 1998; Bennett et al., 1996; Dell'Aglio et al., 2018; Secondi et al., 2012; Silberglied & Taylor, 1978). In addition, as UV sensitivity is not universal among predatory species, UV reflection may offer a route toward targeted or hidden signals only visible to certain observers (Bybee et al., 2012; Cronin & Bok, 2016; Lyytinen et al., 2001). The role of UV components in aposematic signaling is, however, relatively poorly explored.

The color patterns of poison frogs (Dendrobatidae) are incredibly diverse and often display highly contrasting colors and patterning. Visual signals can vary tremendously both between and within species but, despite sampling dozens of populations, reflection of wavelengths outside of the spectrum visible to humans has not been described to date (Hoogmoed & Avila‐Pires, 2012; Roberts et al., 2007; Rojas, 2017; Siddiqi et al., 2004; Twomey et al., 2016; Wang & Shaffer, 2008; Yeager et al., 2012). Intraspecific variation may be discrete or continuous and is found both in sympatry and in allopatry (Rojas, 2017). The function and evolution of this diversity have been the focus of much research, and poison frog color has been variously linked to aposematism, camouflage, and sexual signaling (Crothers & Cummings, 2015; Hegna et al., 2013; Maan & Cummings, 2008; Richards‐Zawacki et al., 2012; Saporito et al., 2007; Summers et al., 1999; Tazzyman & Iwasa, 2010; Willink et al., 2013; Yang et al., 2019). Curiously, however, despite the characterization of aposematic color combinations ranging from red to green, yellow to blue, and black to white, UV reflectance has so far either not been described or not discussed in poison frogs (Barnett et al., 2018; Lawrence & Noonan, 2018; Saporito et al., 2007; Summers et al., 2003), whereas it has been found in aposematic *Heliconius* spp. butterflies that occur in similar habitats and are exposed to similar predators (Bybee et al., 2012; Dell'Aglio et al., 2018; Finkbeiner et al., 2017).

Here, we report the presence of UV‐reflective patterning in an Ecuadorian morph of the poison frog *Oophaga sylvatica* and examine whether UV reflection has a significant role in signal efficacy. Although the visual system of *O. sylvatica* has yet to be studied specifically, inferences from a congener (*O. pumilio*) suggest that UV sensitivity is unlikely (Siddiqi et al., 2004). We therefore focus on the potential antipredator function of the UV signal. To infer specifically if, and how, UV elements could affect contrast between frogs and their habitats, we take a visual modeling approach. We quantified the contribution of UV reflectivity to chromatic and achromatic contrast by comparing between visual models that included and excluded UV sensitivity. To provide additional context, we compared individuals of the UV‐reflecting population and individuals from an allopatric population which shares pattern elements but differs in coloration and lacks UV reflectance.

## 2 METHODS

### 2.1 Photography

**FIGURE 1:**
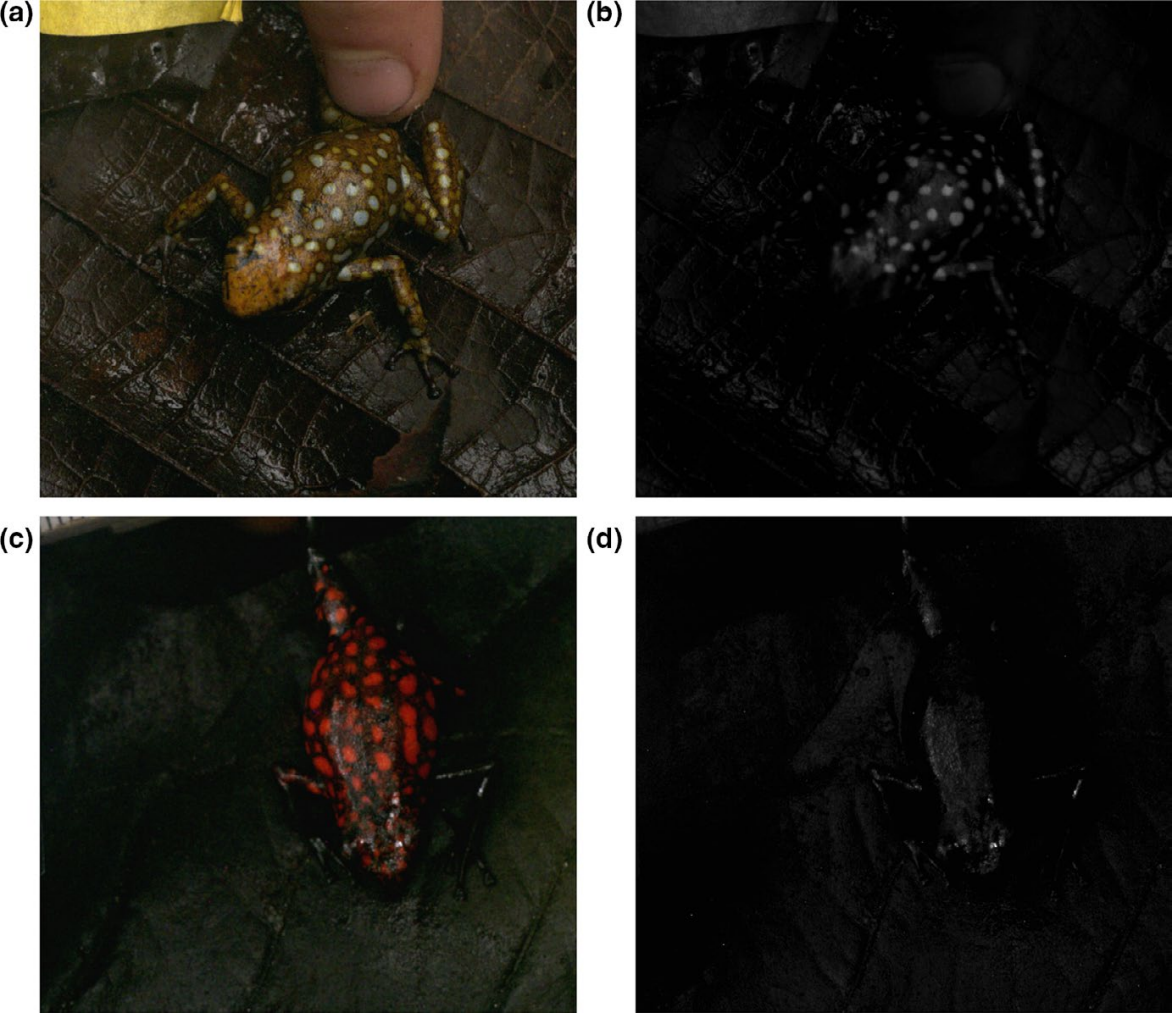
The two morphs of *Oophaga sylvatica* (Top = Lita; Bottom = Perla) photographed in human visible (VIS) light (Left) and UV light (Right). The Lita morph (A = VIS; B = UV, four individuals) is orange with white spots that strongly reflect UV light. The Perla morph (C = VIS; D = UV, four individuals) is black with bright red spots and does not reflect UV light

We sampled *O. sylvatica* from two localities in the Provincia de Esmeraldas in northern Ecuador: near the town of Lita and in the private reserve “Bosque Protector la Perla” outside the city of La Concordia. The Lita morph was orange with UV‐reflective white spots, and the Perla morph was black with red spots (Figure 1). In addition, we photographed a ~140 mm^2^ region of the leaf litter background at the Lita site where the frogs were observed. Digital images were taken using a tripod‐mounted full‐spectrum quartz converted Canon EOS 7D combined with a metal body Nikkor EL 80‐mm lens (known for high UV transmission) and a series of 2” pass filters. To capture the full range of wavelengths visible to UV‐sensitive species, we took two photographs of each frog: one in human visible wavelengths (VIS) and one in the ultraviolet (UV). For human visible spectra, the lens was fitted with a Baader UV‐IR blocking filter that allowed transmission from 420–680 nm. For the UV photographs, we fitted a Baader UV pass filter that allowed transmission from 320–380 nm. To ensure our camera matched known sensitivities included in the Multispectral Image Calibration and Analysis (MICA) Toolbox, the quartz conversion was undertaken at the same facility as the toolbox authors (Troscianko & Stevens, 2015) (Advanced Camera Services Limited). Images were taken in RAW format, and each photograph included 10% and 77% reflectance standards. Downwelling illumination was provided by unfiltered full sun local lighting conditions found within frog microhabitats at the time of peak frog activity. Photograph histograms were manually checked for each photograph (UV and VIS) to ensure proper exposure and prevent data loss due to overexposure. Our sample size was constrained to four individuals due to intense smuggling at the Lita locality, with the most recent event occurring several weeks prior to our sampling. Although few individuals could be sampled, they represent every frog that could be located in the region and is comparable to numbers sampled in similar studies (Cummings et al., 2003).

### 2.2 Image processing

We used the MICA toolbox (Troscianko & Stevens, 2015) in ImageJ v1.52k (Schneider et al., 2012) to linearize and combine our paired VIS and UV photographs into a series of multispectral images. Linearization used the 10% and 77% reflectance standards, and alignment was conducted manually to ensure that small pattern components were accurately overlaid. Regions of interest (ROIs) were created by selecting and combining six patches from the background image and five patches from each component of the frog pattern per photograph: the base colors (Lita = orange; Perla = black) and the spot colors (Lita = UV‐white; Perla = red). Each ROI was placed to avoid regions of specular reflectance.

Each multispectral image was converted into two custom color spaces generated using the UV‐sensitive vision of the Eurasian blue tit (*Cyanistes caeruleus*, Paridae). Although not a natural predator of poison frogs, the blue tit visual system is well characterized and representative of UV‐sensitive birds that are known frog predators (Hart, 2001; Hart et al., 2000; Ödeen & Håstad, 2003). The blue tit is tetrachromatic with single cone peak sensitivity (*λ*_max_) of 573 nm (LWS), 508 nm (MWS), 413 nm (SWS), and 372 nm (UVS), and possesses double cones with λ_max_ of 565 nm (D) (Hart et al., 2000). The single cones are thought to measure chromatic contrast (hue) whereas double cones are thought to measure achromatic (luminance) contrast (Hart et al., 2000; Vorobyev & Osorio, 1998). The single cones of the blue tit populate the retina at a ratio of 1:2:2:3 (UV:SWS:MWS:LWS), and we included Weber fractions of 0.05 to represent intrinsic photoreceptor noise (Hart, 2001; Troscianko & Stevens, 2015; Vorobyev & Osorio, 1998). To assess the contribution of UV reflectance to the saliency of the frog's color pattern we created, and compared between, a UV‐sensitive model and a VIS‐sensitive model. The UV model included the LWS, MWS, SWS, and UVS cones, whereas the VIS model used the LWS, MWS, and SWS cones but excluded the UVS cone. Both models included the D cone as a measure of achromatic contrast.

We calculated chromatic and achromatic contrast as “just noticeable differences” (JNDs) using the receptor noise‐limited visual discrimination model (Vorobyev & Osorio, 1998) in the MICA toolbox (Troscianko & Stevens, 2015; Troscianko & Stevens, 2015; Vorobyev & Osorio, 1998). JNDs represent perceptible contrast relative to the resolution limit set by intrinsic noise within the photon receptor complex. Values < 1 are expected to be imperceptible even under perfect conditions, whereas values > 3 are likely increasingly discriminable under natural variation in lighting conditions (Nokelainen et al., 2019; Vorobyev & Osorio, 1998). For each frog, JNDs of chromatic and achromatic contrast were calculated in a pairwise manner between each ROI: (a) external contrast between the base color and the leaf litter, (b) external contrast between spot colors and the leaf litter background, and (c) internal contrast between the frogs’ base coloration and spot colors.

To assess whether UV reflectance is important in signal saliency we compared achromatic and chromatic contrast between UV‐sensitive and VIS‐sensitive models. If UV reflectance is a major component of signal design, the UV‐sensitive model should report higher contrast for UV‐reflecting regions, whereas the two models should perceive colors lacking UV reflectance in a similar manner.

## 3 RESULTS

**FIGURE 2:**
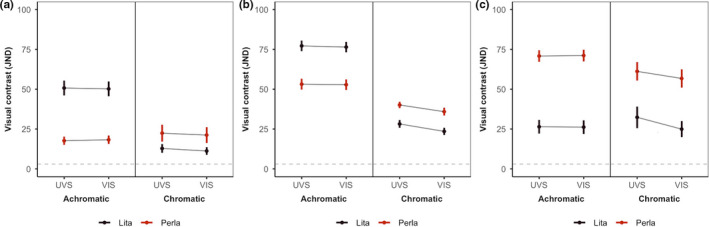
Achromatic and chromatic contrast (mean “just noticeable difference” (JND) ± *SE*, four individuals per locality) for external and internal pattern contrast as viewed by the UV‐sensitive (UVS) and VIS‐sensitive visual models (VIS). (a) External contrast for the base colors against the leaf litter. (b) External contrast for the spot colors against the leaf litter. (c) internal contrast for base colors versus the spot colors. The gray dashed line highlights the conservative visual discrimination threshold (JND = 3) above which colors are increasingly discriminable

We found that for both visual models, the two pattern components of each frog population were highly distinct (well above the conservative visual threshold of 3 JND) from the leaf litter background in both chromatic and achromatic contrast (Figure 2, left & middle; Table 1). Similarly, internal chromatic and achromatic contrast between pattern components was very high for both visual models and both frog populations (Figure 2, right; Table 1). There were minimal differences in achromatic contrast between UV‐sensitive and VIS‐sensitive models for all comparisons. Whereas, there was a trend in all comparisons toward higher chromatic contrast in the UV‐sensitive model (Figure 2; Table 1).

**TABLE 1:** Achromatic and chromatic contrast (mean “just noticeable difference” (JND) ± *SE*) for comparisons between frog and background colors

	UV Model		VIS Model	
	Achromatic	Chromatic	Achromatic	Chromatic
Lita Morph				
External Base	50.77 ± 4.68	12.83 ± 2.76	50.24 ± 4.65	11.24 ± 2.43
External Spot	77.22 ± 3.31	28.21 ± 2.44	76.46 ± 3.31	23.55 ± 2.28
Internal	26.45 ± 4.30	32.39 ± 6.76	26.22 ± 4.29	24.96 ± 5.05
Perla Morph				
External Base	17.67 ± 2.56	22.42 ± 5.31	18.31 ± 2.67	21.23 ± 5.00
External Spot	53.18 ± 3.42	40.13 ± 2.02	52.84 ± 3.33	35.92 ± 2.46
Internal	70.85 ± 3.65	61.22 ± 5.78	71.16 ± 3.67	56.77 ± 5.77

Note

External Base: contrast between the frogs’ base colors and the background; External Spot: contrast between the frogs’ spots and the background; Internal: contrast between the frogs’ base and spot colors. JND values < 1 indicate colors are likely indistinguishable under ideal lighting conditions, colors with values between 1 and 3 are difficult to distinguish under natural conditions, and contrasts with values > 3 are considered distinguishable.

In the Perla frogs, internal achromatic and chromatic contrast was higher than either pattern component was to the leaf litter background (Table 1). Whereas, in the Lita population external contrast, especially achromatic contrast, was higher than internal pattern contrast. In both populations, the spots (Lita = UV‐white; Perla = red) were more distinct from the leaf litter than were the base colors (Lita = orange; Perla = black).

## 4 DISCUSSION

We found that the white spots of the Lita *O. sylvatica* strongly reflect ultraviolet light in contrast to the frogs’ base orange color and the leaf litter background both of which have minimal UV reflectance. The UV‐white spots and the orange base color are highly distinct with high achromatic and chromatic contrast between each other and from the leaf litter background. The Perla *O. sylvatica* similarly has very high internal and external contrast but lacks UV reflectance. UV reflection in Lita frogs, however, has minimal effect on overall visual contrast with the internal and external pattern contrast of both frog populations being high to both UV‐sensitive and VIS‐sensitive visual models. These data suggest that the addition of UV has a negligible impact on signal saliency and begs the question as to why this population of *O. sylvatica* would evolve a UV‐reflective pattern?

The presence of UV‐reflecting spots contained within an aposematic species is not unique to these frogs, with UV‐reflective patterns also being found in certain aposematic butterflies (Bybee et al., 2012; Dell'Aglio et al., 2018; Finkbeiner et al., 2017). Although UV cones are the least abundant single cone within the blue tit retina (Hart, 2001), experiments with captive birds and artificial prey suggest that avian predators can distinguish UV signals and can learn associations between UV reflection and prey profitability (Lyytinen et al., 2001; Werner et al., 2012). Moreover, UV‐reflective marks (“eyespots”) on butterfly wings have been found to deflect bird attacks away from the head but only under lighting conditions representative of dawn or dusk (Olofsson et al., 2010). However, UV signals in aposematic *Heliconius* spp. butterflies have not been found to influence predation risk and instead appear to mediate assortative mate choice (Finkbeiner et al., 2017). As it is improbable that the visual capabilities of *O. sylvatica* include UV wavelengths, it is unlikely that the same applies in this instance (Siddiqi et al., 2004). It is, however, important to further understand the visual capabilities of *O. sylvatica* to confirm that UV signals are not able to influence intraspecific behaviors. Until this is available, the most likely explanation is that UV signals are the result of natural selection or neutral evolutionary processes. While UV signals have not been formally described in poison frogs, they have been documented incidentally. Although UV components were not considered experimentally (or perhaps simply overlooked), it is interesting to note that in *Dendrobates tinctorius* a population displaying UV‐reflective white color patterns performed poorest in predator learning experiments when compared against other conspicuous aposematic morphs (Lawrence & Noonan, 2018).

In both frog populations, we found that chromatic contrast appeared to be slightly higher in the UV‐sensitive model than in the UV‐lacking model, whereas this effect was not evident for achromatic contrast. The visual systems of vertebrates perceive chromatic and achromatic contrast through slightly different pathways. Chromatic contrast is measured through opponent processing, where the visual system compares the relative stimulation of different cone cell types (Kelber & Osorio, 2010). The differences in chromatic contrast for both frog morphs suggest that hue discrimination was generally better in the 4‐dimensional UV‐sensitive model compared to the 3‐dimensional VIS model and that UV reflectance offered minimal increase in saliency. Conversely, achromatic contrast is measured as a single absolute value measured using longwave‐sensitive opsins. In birds, this pigment is contained in double cones, and in humans, the responses of the longwave and medium‐wave cones are averaged together (Osorio & Vorobyev, 2005). This may explain why UV reflection had minimal effect on achromatic signal contrast in Lita *O. sylvatica*. Moreover, the efficacy of aposematic signaling has been linked to both high achromatic and chromatic contrast (Aronsson & Gamberale‐Stille, 2009; Gamberale‐Stille, 2001; Halpin et al., 2020; Prudic et al., 2006; Stevens et al., 2010). This phenomenon may, therefore, underlie an apparent abundance of longwave colors (i.e., red and yellow) rather than shortwave colors (i.e., blue and UV) in aposematic signals.

Furthermore, color in poison frogs is under genetic control and is produced by the interaction between melanin and carotenoids pigments that selectively absorb certain wavelengths of light and structural colors that reflect and scatter light (Rodríguez et al., 2020; Stuckert et al., 2019). The color white results from the reflection of all visible wavelengths with brighter whites requiring more efficient reflection. It stands to reason that as there is no firm boundary between human (or *O. sylvatica*) visible and UV wavelengths, compounds that increase reflectivity across all wavelengths between 400 and 700 nm will also include significant reflection below 400 nm. Indeed, such an effect may be particularly pronounced when reflectance is maximized close to the limits of visual perception. For example, although frogs are not known to possess dedicated UV‐sensitive cones (Donner & Yovanovich, 2020), male Balkan moor frogs (*Rana arvalis wolterstorffi*) turn bright UV‐blue in the breeding season in order to signal to conspecifics (Ries et al., 2008). Incidental, nonfunctional, UV reflectance may therefore be most common in bright blue or white color patterns.

In general, it is likely that our anthropogenic bias leads us to predict that the addition of UV wavelengths, which are not perceivable to us, must be a private channel or highly important additions to an aposematic signal. However, selection for non‐UV signals, visible to conspecifics and heterospecifics alike, may simply produce a UV signature as a by‐product. As such, the reflection of UV wavelengths is not necessarily important to the overall signal (Kevan et al., 2001; Stevens & Cuthill, 2007). Moreover, as the signal is highly salient across many wavelengths, it is unlikely that UV reflectance subsequently would be selected against, perhaps opening the possibility that UV signals evolve neutrally.

In conclusion, the most parsimonious explanation for UV reflectivity in this aposematic visual signal is an extension of the already highly salient broad‐spectrum reflectance of the white spots rather than a separate or additional signal component. UV reflectance is likely a by‐product, the result of selection for high internal and external contrast across all wavelengths visible to both conspecifics and heterospecifics. However, the presence of UV reflection in *O. sylvatica* demonstrates that we cannot safely ignore UV colors when studying predation risk and signaling in aposematic species. In poison frogs, this is especially true as visual sensitivity has only been characterized for a single species. Further work is necessary to understand the prevalence of UV reflection in aposematic species and how UV‐sensitive predators and conspecifics may respond to such signals in the wild.

## CONFLICT OF INTEREST

None declared.

## AUTHOR CONTRIBUTIONS

**Justin Yeager:** Conceptualization (equal); data curation (equal); formal analysis (equal); funding acquisition (equal); investigation (equal); methodology (equal); project administration (equal). **James B. Barnett:** Formal analysis (equal); investigation (equal); methodology (equal); project administration (equal); writing – review and editing (equal).

## Data Availability

Data have been deposited into Dryad https://doi.org/10.5061/dryad.s7h44j14x
